# Complete Genome Sequence of *Burkholderia* sp. Strain RPE64, Bacterial Symbiont of the Bean Bug *Riptortus pedestris*

**DOI:** 10.1128/genomeA.00441-13

**Published:** 2013-07-05

**Authors:** Tomoko F. Shibata, Taro Maeda, Naruo Nikoh, Katsushi Yamaguchi, Kenshiro Oshima, Masahira Hattori, Tomoaki Nishiyama, Mitsuyasu Hasebe, Takema Fukatsu, Yoshitomo Kikuchi, Shuji Shigenobu

**Affiliations:** National Institute for Basic Biology, Okazaki, Japana; Department of Liberal Arts, the Open University of Japan, Chiba, Japanb; Center for Omics and Bioinformatics, Graduate School of Frontier Sciences, University of Tokyo, Kashiwa, Japanc; Advanced Science Research Center, Kanazawa University, Kanazawa, Japand; Department of Basic Biology, School of Life Science, Graduate University for Advanced Studies, Okazaki, Japane; Bioproduction Research Institute, National Institute of Advanced Industrial Science and Technology, Tsukuba, Japanf

## Abstract

We isolated *Burkholderia* symbiont strain RPE64 from the bean bug *Riptortus pedestris*. Analysis of the complete 6.96-Mb genome, which consists of three chromosomes and two plasmids, will facilitate further understanding of insect–microbe symbiosis and the development of pest-control technologies.

## GENOME ANNOUNCEMENT

The bean bug *Riptortus pedestris*, a leguminous-crop pest, is specifically associated with a betaproteobacterial *Burkholderia* symbiont in the posterior midgut (1). *R. pedestris* acquires the *Burkholderia* symbiont from the soil every generation (2), and the symbiont infection enhances growth and body size of the host insect (3). Here, we analyzed the whole-genome sequence of *Burkholderia* sp. strain RPE64, isolated from field-collected *R. pedestris* (4).

For whole-genome shotgun sequencing of the *Burkholderia* symbiont, we used paired-end sequencing with HiSeq 2000 (Illumina) and long sequencing with PacBio RS (Pacific Biosciences). Four Illumina libraries, with 180-, 300-, 500-, and 800-bp insertions, were constructed and sequenced 101 bp from both ends, yielding 32.6 Gb of raw data (accession no. DRA000987). After the removal of adaptor sequences and low-quality reads (quality value of <20), 25.6 Gb of high-quality sequences remained. An approximately-6-kb insert PacBio library was constructed and sequenced, yielding 189,571 reads with 2.1 to 2.7 kb mean maximum subread length, totaling 449 Mb of independent fragment reads (accession no. DRA000982). The Illumina and PacBio reads were assembled *de novo* using a hybrid assembly algorithm implemented in Allpaths-LG software (5), yielding 24 contigs. PacBio, which is tolerant to G+C bias, complemented the low coverage of the Illumina data in high-G+C regions. While scaffolds assembled from the Illumina reads contained 11,407 ambiguous bases (i.e., Ns), adding the PacBio reads resolved all but three Ns. Gaps between contigs were closed by Sanger sequencing of PCR-amplified fragments. Coding genes were predicted with Glimmer (6) and annotated by a BLAST search against UniProt.

The complete genome of *Burkholderia* sp. strain RPE64 is 6.96 Mb and comprises three circular chromosomes and two plasmids: chromosome 1 (3,013,410 bp, 2,907 protein-coding sequences [CDSs]), chromosome 2 (1,465,356 bp, 1,422 CDSs), chromosome 3 (900,830 bp, 853 CDSs), plasmid 1 (1,275,199 bp, 1,222 CDSs), and plasmid 2 (309,692 bp, 328 CDSs). The G+C content is 60.1% to 63.5%.

The closest-sequenced-genome relative of strain RPE64 is the soil-isolated *Burkholderia* sp. strain YI23. The 16S rRNA sequences have high nucleic acid identity (98.6%) but different overall genomic structures; the total genome size of RPE64 is 1.93 Mb smaller than that of YI23. RPE64 lacks a plasmid equivalent to BYI23_F of YI23, where fenitrothion-degrading genes (*mhqA* and *mhqB*) are located. Consistent with the absence of these genes, RPE64 cannot degrade the insecticide.

While some genomes of human-pathogenic and plant-associated *Burkholderia* isolates have been sequenced (7), this is the first complete genome sequence of an insect-associated symbiotic *Burkholderia* strain. Together with the transcriptome data of the host midgut crypts (8), the complete sequence of this symbiotic *Burkholderia* strain will contribute to the molecular understanding of the insect–microbe symbiosis. We recently found that fenitrothion-degrading strains of symbiotic *Burkholderia* confer resistance of the host insect to fenitrothion and other organophosphorus insecticides (9). The RPE64 genome provides a good reference for comparatively analyzing the mechanisms of symbiont-mediated insecticide resistance.

### Nucleotide sequence accession numbers.

The complete genome sequence of *Burkholderia* sp. strain RPE64 (including three chromosomes and two plasmids) has been deposited in DDBJ/EMBL/GenBank under accession no. AP013058 to AP013062.

## References

[1] Kikuchi Y Meng XY Fukatsu T 2005 Gut symbiotic bacteria of the genus *Burkholderia* in the broad-headed bugs *Riptortus clavatus* and *Leptocorisa chinensis* (Heteroptera: Alydidae). Appl. Environ. Microbiol. 71:4035–4043 16000818 10.1128/AEM.71.7.4035-4043.2005 PMC1169019

[2] Kikuchi Y Hosokawa T Fukatsu T 2011 Specific developmental window for establishment of an insect-microbe gut symbiosis. Appl. Environ. Microbiol. 77:4075–4081 21531836 10.1128/AEM.00358-11 PMC3131632

[3] Kikuchi Y Hosokawa T Fukatsu T 2007 Insect-microbe mutualism without vertical transmission: a stinkbug acquires a beneficial gut symbiont from the environment every generation. Appl. Environ. Microbiol. 73:4308–4316 17483286 10.1128/AEM.00067-07 PMC1932760

[4] Kikuchi Y Hosokawa T Fukatsu T 2011 An ancient but promiscuous host-symbiont association between *Burkholderia* gut symbionts and their heteropteran hosts. ISME J. 5:446–460 20882057 10.1038/ismej.2010.150 PMC3105724

[5] Ribeiro FJ Przybylski D Yin S Sharpe T Gnerre S Abouelleil A Berlin AM Montmayeur A Shea TP Walker BJ Young SK Russ C Nusbaum C MacCallum I Jaffe DB 2012 Finished bacterial genomes from shotgun sequence data. Genome Res. 22:2270–2277 22829535 10.1101/gr.141515.112 PMC3483556

[6] Delcher AL Bratke KA Powers EC Salzberg SL 2007 Identifying bacterial genes and endosymbiont DNA with Glimmer. Bioinformatics 23:673–679 17237039 10.1093/bioinformatics/btm009 PMC2387122

[7] Winsor GL Khaira B Van Rossum T Lo R Whiteside MD Brinkman FS 2008 The *Burkholderia* genome database: facilitating flexible queries and comparative analyses. Bioinformatics 24:2803–2804 18842600 10.1093/bioinformatics/btn524 PMC2639269

[8] Futahashi R Tanaka K Tanahashi M Nikoh N Kikuchi Y Lee BL Fukatsu T 2013 Gene expression in gut symbiotic organ of stinkbug affected by extracellular bacterial symbiont. PLoS One 8:e64557 23691247 10.1371/journal.pone.0064557 PMC3653873

[9] Kikuchi Y Hayatsu M Hosokawa T Nagayama A Tago K Fukatsu T 2012 Symbiont-mediated insecticide resistance. Proc. Natl. Acad. Sci. U. S. A. 109:8618–8622 22529384 10.1073/pnas.1200231109 PMC3365206

